# Identification of LRP1^+^CD13^+^ human periosteal stem cells that require LRP1 for bone repair

**DOI:** 10.1172/jci.insight.173831

**Published:** 2024-11-22

**Authors:** Youngjae Jeong, Lorenzo Deveza, Laura Ortinau, Kevin Lei, John R. Dawson, Dongsu Park

**Affiliations:** 1 Department of Molecular and Human Genetics and; 2 Department of Orthopedic Surgery, Baylor College of Medicine, Houston, Texas, USA.

**Keywords:** Bone biology, Stem cells, Adult stem cells, Human stem cells

## Abstract

Human periosteal skeletal stem cells (P-SSCs) are critical for cortical bone maintenance and repair. However, their in vivo identity, molecular characteristics, and specific markers remain unknown. Here, single-cell sequencing revealed human periosteum contains SSC clusters expressing known SSC markers, podoplanin (*PDPN*) and *PDGFRA*. Notably, human P-SSCs, but not bone marrow SSCs, selectively expressed identified markers low density lipoprotein receptor-related protein 1 (LRP1) and CD13. These LRP1^+^CD13^+^ human P-SSCs were perivascular cells with high osteochondrogenic but minimal adipogenic potential. Upon transplantation into bone injuries in mice, they preserved self-renewal capability in vivo. Single-cell analysis of mouse periosteum further supported the preferential expression of LRP1 and CD13 in *Prx1*^+^ P-SSCs. When *Lrp1* was conditionally deleted in *Prx1* lineage cells, it led to severe bone deformity, short stature, and periosteal defects. By contrast, local treatment with an LRP1 agonist at the injury sites induced early P-SSC proliferation and bone healing. Thus, human and mouse periosteum contains unique osteochondrogenic stem cell subsets, and these P-SSCs express specific markers, LRP1 and CD13, with a regulatory mechanism through LRP1 that enhances P-SSC function and bone repair.

## Abstract 2

The discovery of human periosteum-resident skeletal stem cells with novel LRP1 and CD13 marker expression and their osteochondrogenic potentials

## Introduction

Tissue-resident skeletal stem cells (SSCs) are essential for bone growth and lifelong bone regeneration and repair in humans. Adult SSCs are highly heterogeneous and present in the bone marrow (BM) and periosteum (outer bone layer). In many orthopedic procedures, removal of the periosteum generally interrupts fracture repair, implying that periosteal skeletal stem cells (P-SSCs) play a critical role in fracture healing and regeneration (1–3). For these reasons, the application of bone grafts with P-SSCs is considered an ideal therapeutic candidate for nonunion fractures, bone defects, and bony fusions (4). Recently, multiple subsets of mouse skeletal stem and progenitor cells were discovered by utilizing transgenic tools, and many studies have focused on the distinct functions and markers of mouse P-SSCs (5–13). While the presence of similar stem/progenitor cells in the human periosteum has been proposed, the in vivo identity and function of endogenous human P-SSCs are far more elusive. In fact, whether such heterogeneity exists within human P-SSC populations and how they differ from BM-SSCs remain unknown.

To dissect cellular compositions within SSCs, single-cell RNA sequencing (scRNA-Seq) has been used to identify and characterize cellular heterogeneity in the BM stromal population in mice and humans (2, 3, 14–17). These comprehensive analyses of BM stromal cells (BMSCs) improved our understanding of their diversity and regulatory roles in the maintenance of both the skeletal and hematopoietic system. The first embryonic skeletogenesis cell atlas of human bones was generated from human embryonic long bone and calvaria and revealed the presence of multiple subsets of stem/progenitor cells and their lineage hierarchy during embryonic skeletal development (4). Other in vitro studies revealed that freshly isolated human BM aspirate cells fail to meet the criteria for cultured mesenchymal stem cells, suggesting the presence of heterogeneity and distinct gene expression profiles for endogenous SSCs (14, 17). A recent scRNA-Seq study with neonatal human BM cells identified 7 subsets of human skeletal stem/progenitor cells (SSPCs) with differential expression of podoplanin (PDPN), CD146, CD73, CD164, and THY1 (1). While previous studies allowed us to define multiple subsets of SSPCs, a main limitation is that these studies only used BM cells. Therefore, the cellular heterogeneity and molecular signatures of human periosteal cells and their SSC subsets remain elusive. Furthermore, the distinct markers of endogenous human P-SSCs are essentially unknown.

Here, we define for the first time to our knowledge the cellular composition and heterogeneity of the human periosteum by single-cell analysis. We also define the molecular characteristics of endogenous human P-SSCs and identify potentially novel human P-SSC–specific markers, low density lipoprotein receptor-related protein 1 (LRP1) and CD13. In addition, we report that the combination of LRP1 and CD13 with other SSC markers (PDPN and PDGFRA) can distinguish and isolate human and mouse P-SSCs with osteochondrogenic differentiation capacity and that LRP1 plays a critical role in skeletal development and periosteal progenitor cell regulation.

## Results

### Single-cell analysis reveals distinct human P-SSC clusters with known SSC marker expression.

**Figure 1:**
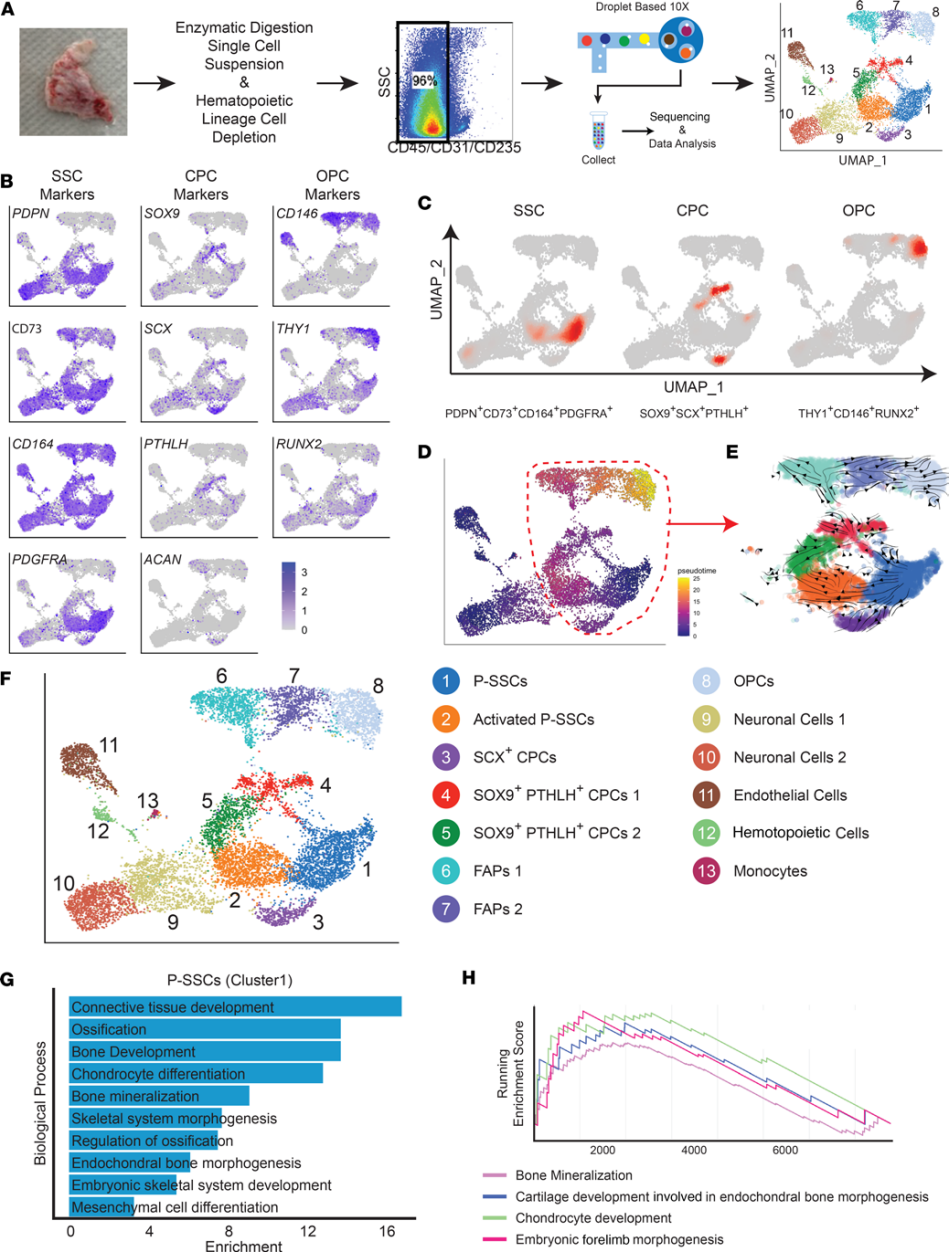
Single-cell analysis reveals human periosteal SSC clusters with known SSC marker expression. (**A**) Schematic process of human periosteal stromal cell isolation and uniform manifold approximation and projection (UMAP) plot of total scRNA-Seq data (10x Genomics) of FACS-sorted CD45*^–^*CD31*^–^*CD235a*^–^* periosteal cells from the indicated donors. (**B**) UMAP-based transcriptional plots of representative human SSC, chondrogenic progenitor cell (CPC), and osteogenic progenitor cell (OPC) markers. (**C**) UMAP joint density plots showing the distribution of indicated SSC, CPC, and OPC markers. (**D** and **E**) Pseudotime (**D**) and RNA velocity (**E**) visualized on the UMAP of the periosteal stromal cell population. (**F**) UMAP visualization of 13 color-coded clusters. (*n* = 10,920.) (**G**) Enriched biological process Gene Ontology (GO) terms of differentially expressed genes (DEGs) in the P-SSC cluster (cluster 1). (**H**) Positive running enrichment score in the P-SSC cluster.

The human periosteum is a thin fibrous layer composed of various cells with different biological properties, but little is known about the cellular identity and regulatory mechanism of the human periosteum-resident cells and their stem cells. To define the cellular composition of human periosteal cells, we obtained 4 sets of intact (uninjured but discarded) periosteal specimens from the medial malleoli and distal radius of patients during fracture repair surgery (Supplemental Figure 1A; supplemental material available online with this article; https://doi.org/10.1172/jci.insight.173831DS1). Immediately after specimen collection, we performed enzymatic digestion, hematopoietic lineage depletion to enrich stroma cell populations, and isolation of nonhematopoietic/nonendothelial (CD45*^−^*CD31*^−^*CD235a*^−^*) periosteal cells using fluorescence-activated cell sorting (FACS). Sorted cells were then used for droplet-based 10x Genomics Chromium scRNA-Seq. The datasets were filtered to remove cells with fewer than 500 detected unique molecular identifiers (UMIs) and genes, leaving a total of 10,920 single cells from 4 periosteal samples. The comparison of 4 periosteal datasets revealed that there was no substantial batch effect after the integration of datasets by Seurat (18) (Figure 1A and Supplemental Figure 1B). Subsequent UMAP clustering showed that the periosteal cells comprised at least 13 distinct clusters, supporting that endogenous periosteal cells are highly heterogeneous (Figure 1A). To characterize each of these clusters, we next performed cell type annotation analysis with the previously known markers and cell type–specific gene signatures. We found 8 clusters (cluster 1 to 8) grouped together with expression of mesenchymal lineage markers, and 5 other clusters distinctly separated with expression of NCAM^+^STMN1^+^ neuronal markers (clusters 9 and 10), CD31^+^ endothelial cell markers (cluster 11), CD45^+^ hematopoietic cell markers (cluster 12), or CD14^+^CD11b^+^ monocyte/macrophage markers (cluster 13) (Figure 1, B and F, and Supplemental Figure 2).

We next determined which of these clusters annotate P-SSCs and osteochondrogenic progenitor cells. Given there were no specific human P-SSC markers, we first analyzed the expression of osteogenic and chondrogenic progenitor markers as well as previously reported human BM-SSC markers (1). Notably, we found that key chondrogenic factors (*SOX9*, *SCX*, *PTHLH*, *ACAN*) were expressed in clusters 3, 4, and 5 and key transcription factors (*MSX2* and *HEY1*) in cluster 3 (Figure 1, B and C, and Supplemental Figure 2E). By contrast, cluster 8 selectively expressed early osteoprogenitor (OPC) markers and transcription factors (*CD146*, *THY1*, *RUNX2*, and *DLX5*) with few mature osteoblast markers, suggesting that cluster 8 represented osteogenic progenitors (Figure 1, B and C, and Supplemental Figure 2F). Notably, overlaying of multiple human BM SSC surface markers (*PDPN*, *CD73*, *CD164*, and *PDGFRA*) showed their enrichment in cluster 1 (Figure 1C). Since SSCs supply diverse cell types during bone development and possess multilineage differentiation potential, we used lineage trajectory (Monocle) and RNA velocity dynamics (scVelo) to define the mesenchymal subpopulation splicing kinetics and to identify each cell’s position during the differentiation processes (19–21). Nonrelevant clusters (clusters 9–13) were excluded from the analysis to minimize the misinterpretation of relationships between cells, lineage trajectory, and RNA velocity. These data consistently showed the transition of cellular state from P-SSCs (cluster 1) to chondrogenic progenitor cells (CPCs) (cluster 3, 4, and 5), fibro-adipogenic progenitors (FAPs) (cluster 6 and 7), and OPCs (cluster 8), supporting that there is a multipath trajectory potential from SSCs in human periosteum (22) (Figure 1, D and E).

In addition, cell cycle analysis revealed that the P-SSC cluster (cluster 1) selectively expressed stemness markers (*SOX4*, *GAS1*, and *DPP4*) with the highest G1 population among mesenchymal lineage cells, while SOX9^+^PTHLH^+^ CPCs 1 (cluster 4) contained the highest G2M phase (Supplemental Figure 3). Consistent with lineage trajectory and RNA velocity analysis, Gene Ontology (GO) analysis showed the P-SSC cluster highly expressed genes involved in skeletal system development, chondrocyte differentiation, ossification, and endochondral bone morphogenesis, supporting that this cluster encompasses stem and progenitor cells with multilineage differentiation potentials (Figure 1G). Further, the P-SSC cluster showed a positive enrichment score for bone mineralization, endochondral bone morphogenesis, and chondrocyte development (Figure 1H).

Recently, several markers of P-SSCs have been identified in murine models. However, whether human P-SSCs similarly express mouse P-SSC markers has not been elucidated. We therefore analyzed the expression of murine P-SSC markers in the UMAP plots and found *CTSK* (a recently identified mouse P-SSC marker) (8), was highly expressed in cluster 1, while *PRRX1* (a marker for limb bud development and periosteal progenitor cells) (12, 23) was expressed in clusters 1–10 (Supplemental Figure 4). Further, cluster 1 expressed other BM perivascular SSC markers (*CXCL12* and *NES*), suggesting these clusters annotate periosteal SSPCs, which are highly similar to mouse P-SSCs and their stem cell marker expression (Supplemental Figure 3). We also verified that fully differentiated cell markers were not expressed in these clusters (Supplemental Figure 2, E–G). Taken together, these data suggest that the human periosteum contains a PDPN^+^CD73^+^CD164^+^PDGFRA^+^ P-SSC cluster (cluster 1) distinct from SOX9^+^SCX^+^PTHLH^+^ and SOX9^+^SCX^+^PTHLH*^−^* chondroprogenitors (cluster 3 and 4) and THY1^+^CD146^+^RUNX2^+^ osteoprogenitor cluster (cluster 8).

### Human P-SSCs express LRP1 and CD13 as P-SSC surface markers.

**Figure 2:**
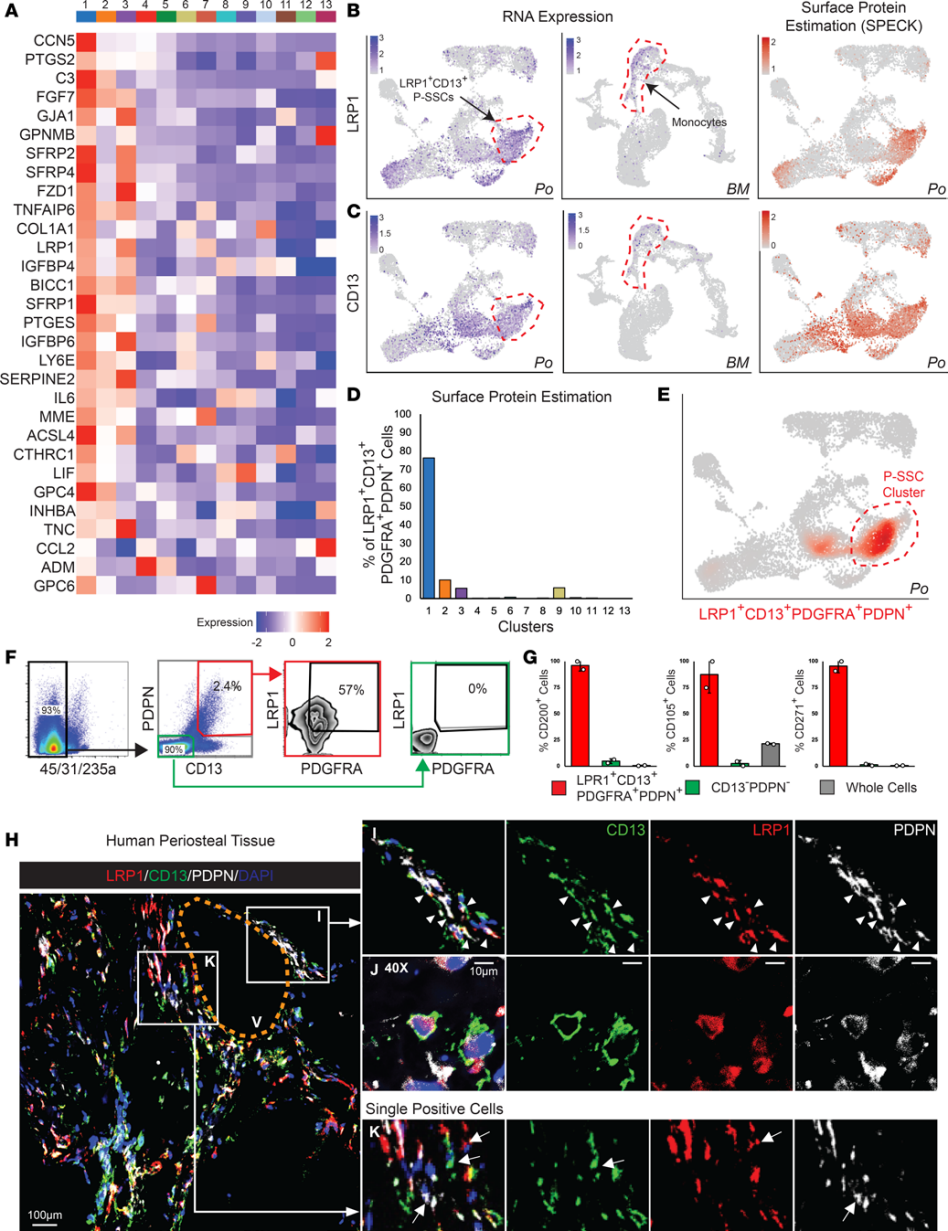
Human P-SSCs highly express LRP1, and the combination of LRP1 and SSC markers, CD13^+^PDPN^+^PDGFRA^+^, can specify P-SSC clusters. (**A**) Heatmaps showing the top 30 DEGs from plasma membrane protein complex in P-SSCs. (**B** and **C**) Visualization of periosteal (left) and BM+BM aspirate concentrate (right) cells with UMAP plots showing LRP1 and CD13 gene expression (**B**) and periosteal surface protein estimation of LRP1 and CD13 expression by SPECK (Surface protein abundance Estimation using CKmeans-based clustered thresholding) method (**C**). (**D**) Percentage distribution of LRP1^+^CD13^+^PDGFRA^+^PDPN^+^ surface protein estimation on each periosteal cluster. (**E**) LRP1^+^CD13^+^PDGFRA^+^PDPN^+^ cells on the periosteal cell UMAP plot. (**F**) FACS analysis of periosteal cell surface expression of LRP1 and PDGFRA in CD13^+^PDPN^+^ and CD13*^−^*PDPN*^−^*. (**G**) The surface expression of the indicated SSC markers (CD200, CD105, and CD271) in LRP1^+^CD13^+^PDGFRA^+^PDPN^+^, CD13*^−^*PDPN*^−^*, and whole cells from the periosteum. (**H**–**K**) Representative immunofluorescence staining images of the indicated markers (CD13, LRP1, and PDPN) on periosteal tissue at 20× (**H** and **I**) and 40× (**J**) original magnification (V, blood vessel). Periosteal cell coexpressing CD13 (green), LRP1 (red), and PDPN (white) are indicated with arrowheads (**I**), and single-positive cells are indicated with arrow (**K**).

The characteristics of human P-SSCs remain largely undefined because of the lack of knowledge regarding their specific markers. Therefore, we next examined the DEGs and cell surface markers in the P-SSC cluster (cluster 1) compared with other clusters. Among the top genes, we filtered out plasma membrane expressing genes that were not detectable in scRNA-Seq datasets of freshly isolated human BM from femoral neck and BM aspirate cells (14). Interestingly, we observed that *LRP1* and *CD13* transcripts were highly expressed in P-SSC and progenitor clusters (Figure 2, A and B). Subsequent UMAP clustering of periosteal and BM aspirate cells revealed *LRP1* and *CD13* expression were not detectable in BM stromal cells, though monocyte subsets showed low expression (Figure 2B). Further, surface protein expression estimation by SPECK (Surface Protein abundance Estimation using CKmeans-based clustering thresholding) showed more distinct and stronger *LRP1* and *CD13* expression in the P-SSC cluster (Figure 2C), while its expression was absent in the BM dataset. When surface protein estimation of high *LRP1*- and *CD13*-expressing cells (*LRP1* > 1) was combined with *PDPN* and *PDGFRA* (known SSPC markers) (1, 24–26), we found that approximately 76% of LRP1^+^CD13^+^PDGFRA^+^PDPN^+^ cells were enriched in P-SSCs (cluster 1) (Figure 2, D and E).

Next, to test whether the LRP1^+^CD13^+^PDGFRA^+^PDPN^+^ combination could be used to isolate human P-SSCs, we performed FACS analysis of cells from human periosteum tissue and found that 2.4% (2.2 ± 0.5, *n* = 3) of CD45*^−^*CD31*^−^*CD235a*^−^* periosteal cells were CD13^+^PDPN^+^ and approximately 57% of CD13^+^PDPN^+^ periosteal cells were LRP1^+^PDGFRA^+^ (Figure 2F, red boxes). However, none of the CD13*^−^*PDPN*^−^* cells expressed LRP1 and PDGFRA (Figure 2F, green boxes). More importantly, most of the LRP1^+^CD13^+^PDGFRA^+^PDPN^+^ periosteal cells expressed SSC immunotypic markers, namely CD200 (~96%), CD105 (~88%), and CD271 (~96%), while only a small subset (<5%) of CD13*^−^*PDPN*^−^* cells expressed CD200, CD105, and CD271, signifying the specificity of LRP1 in human P-SSCs (Figure 2G). To further validate the coexpression of LRP1, CD13, and PDPN in human P-SSCs and their tissue locations, we performed immunofluorescence staining of LRP1, CD13, and PDPN on human periosteal tissues and found LRP1, CD13, and PDPN expression colocalized in a subset of periosteal cells (Figure 2, H–K). We also found that these LRP1^+^CD13^+^ P-SSCs preferentially resided in a perivascular location (Figure 2, H–K). Taken together, these data suggest LRP1 and CD13 are highly expressed in human P-SSC clusters, and the combination of LRP1 and CD13 with PDPN and PDGFRA can identify endogenous human P-SSCs with high specificity.

### LRP1 and CD13 are selective markers that can distinguish human P-SSCs from BMSCs.

**Figure 3:**
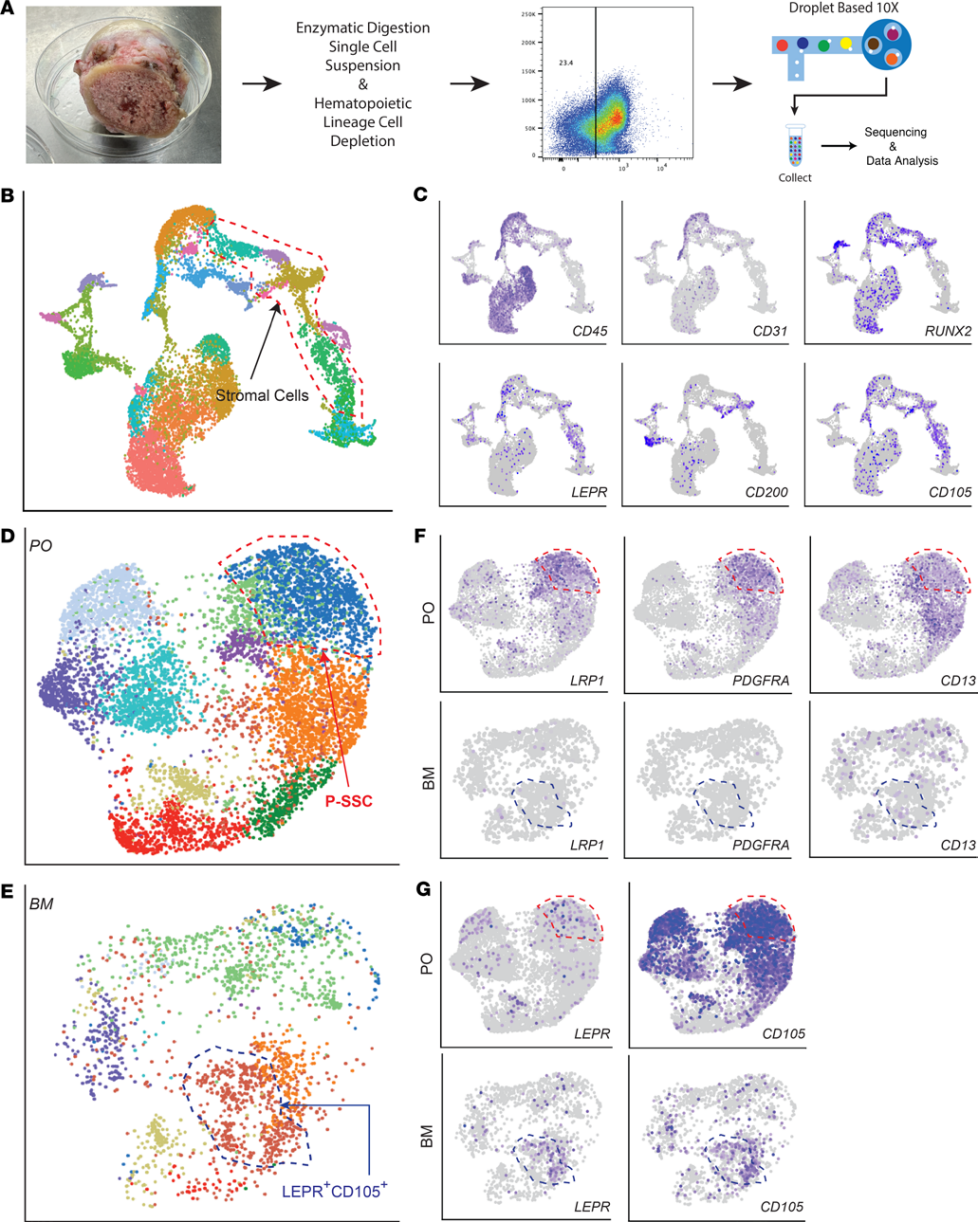
Human P-SSCs distinctly express LRP1 and SSC markers compared with BM SSCs. (**A**) Schematic process of human BM cell isolation and UMAP plot of total scRNA-Seq data (10x Genomics) of hematopoietic lineage–depleted and FACS-sorted CD45*^−^*CD31*^−^*CD235a*^−^* BM cells. (**B** and **C**) UMAP plot of BM combined with BM aspirate concentrate (**B**) and human hematopoietic/endothelial (CD45 & CD31) and stromal cell (RUNX2, LEPR, CD200, & CD105) markers (**C**). (**D** and **E**) Integrated UMAP plot of periosteal (**D**) and BM (**E**) cells with 2 distinct minimally overlapping clusters. (**F**) Selective expression of LRP1, PDGFRA, and CD13 in P-SSCs (top row: P-SSCs; bottom row: BMSCs). (**G**) UMAP plot of BM skeletal progenitor markers (top row: P-SSCs; bottom row: BMSCs).

SSCs in different bone compartments have distinct functions and site-specific regulation. However, it is not known how human P-SSCs differ from BMSCs at the single-cell level. Furthermore, selective markers for human P-SSCs are not well elucidated. Given scRNA-Seq data from freshly isolated BM aspirate concentrates were recently reported and publically available (14), we combined these BM aspirate datasets with our BM single-cell datasets from femoral neck and compared these BM cell datasets with our periosteal cell datasets (Figure 3A). After selecting high-quality single cells, we first defined BMSC subsets (CD45*^−^*CD31*^−^*LEPR^+^CD105^+^ and CD45*^−^*CD31*^−^*RUNX2^+^CD200^+^) in BM cells through UMAP clustering analysis (Figure 3, B and C). Next, the integration of BM cell subsets with periosteal cell subsets revealed some human periosteal cell clusters overlapped with BM stromal clusters. However, the human P-SSCs and BMSCs (LEPR^+^CD105^+^) formed distinct clusters, suggesting differential molecular signatures of human P-SSC clusters compared with BMSC clusters (Figure 3, D, E, and G). More importantly, we determined that *LRP1*, *CD13*, and *PDGFRA* transcripts were highly expressed in P-SSC clusters but nearly undetectable in BMSCs (Figure 3F). These results suggest that the LRP1 and CD13 combination can be a selective marker for endogenous human P-SSCs (14).

### LRP1^+^CD13^+^PDGFRA^+^PDPN^+^ human P-SSCs are osteochondrogenic and long-term repopulating cells in vitro.

**Figure 4:**
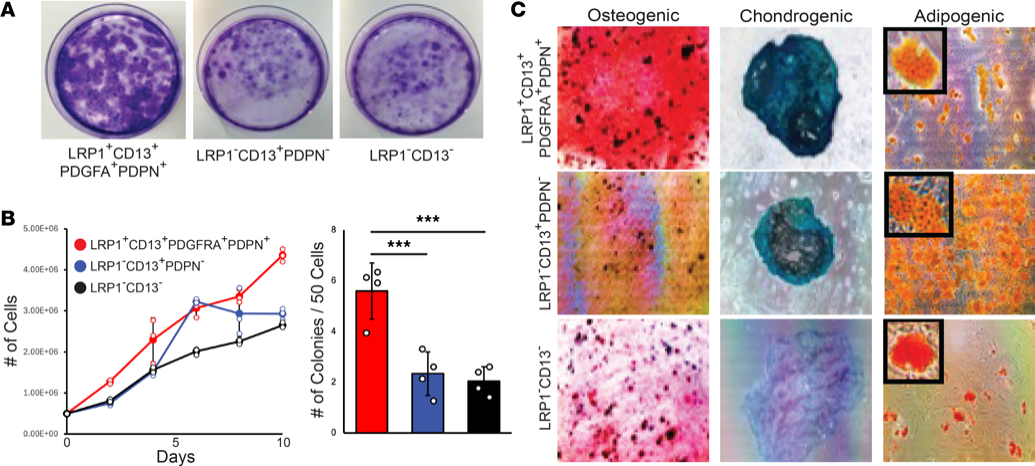
LRP1^+^CD13^+^PDGFRA^+^PDPN^+^ human periosteal cells are highly clonogenic and possess multipotent differentiation capacity in vitro. (**A**) LRP1^+^CD13^+^PDGFRA^+^PDPN^+^ cells have a higher colony number with more viable cells than LRP1*^−^*CD13^+^PDPN*^−^* or LRP1*^−^*CD13*^−^* cells. (**B**) Quantitative measurement of the clonogenic assay shows that LRP1^+^CD13^+^PDGFRA^+^PDPN^+^ cells have ~2-fold more cells than LRP1*^−^*CD13^+^PDPN*^−^* and LRP1*^−^*CD13*^−^* cells at day 10 (*P* < 0.001). (**C**) Comparison of in vitro trilineage differentiation of LRP1^+^CD13^+^PDGFRA^+^PDPN^+^, LRP1*^−^*CD13^+^PDPN*^−^*, and LRP1*^−^*CD13*^−^* cells in osteogenic (alizarin red), chondrogenic (Alcian blue), and adipogenic culture medium (Oil Red O). Data presented as mean ± SD. ***P < 0.001 by 2-tailed Student’s *t* test.

**Figure 5:**
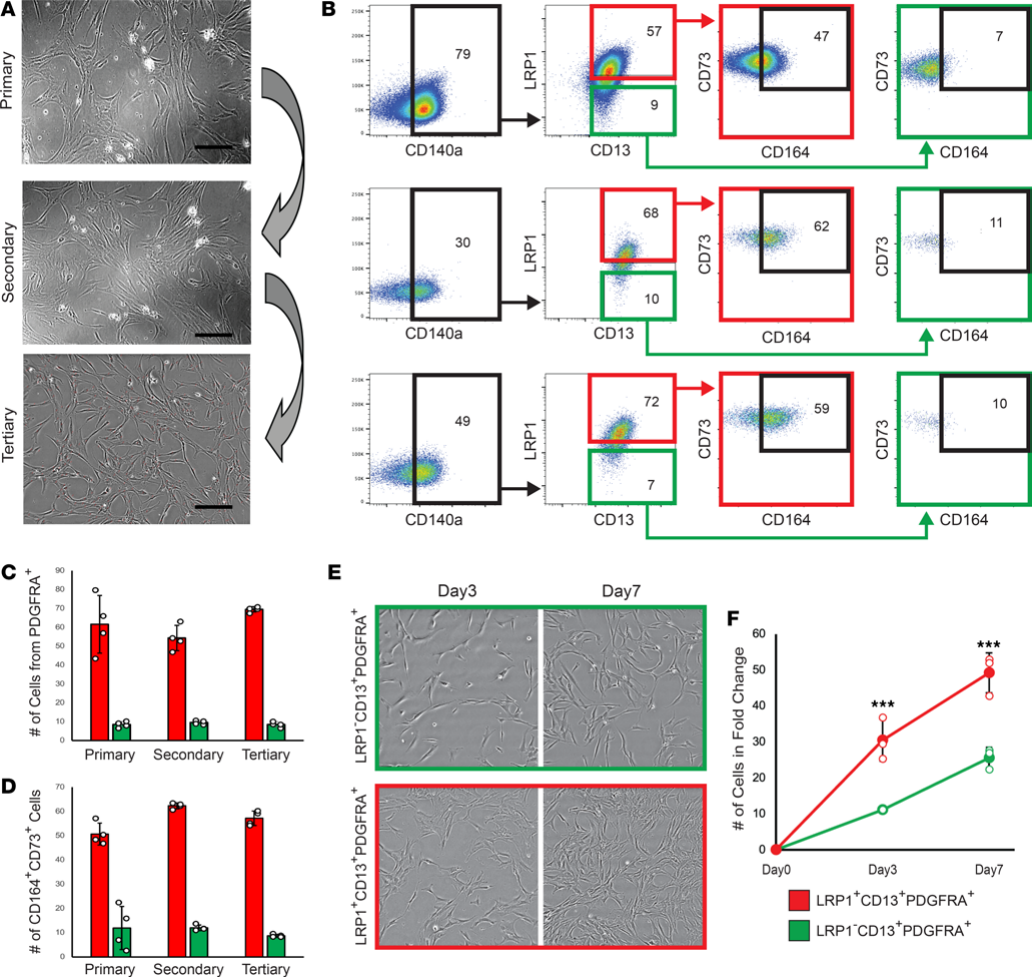
LRP1^+^CD13^+^PDGFRA^+^PDPN^+^ human periosteal cells can self-renew and maintain SSC marker expression in vitro. (**A**) Images showing primary, secondary, and tertiary periosteal cell colonies. 10× original magnification. (**B**) FACS plots show that LRP1^+^CD13^+^PDGFRA^+^ cells are maintained over multiple passages and show SSC surface marker expression (CD164 and CD73), while LRP1*^−^*CD13^+^PDGFRA^+^ cells lack SSC surface marker expression. (**C** and **D**) FACS analysis of cell surface expression of LRP1^+^CD13^+^ cells from PDGFRA^+^ cells (**C**) and CD164^+^CD73^+^ cells from LRP1^+^CD13^+^PDGFRA^+^ and LRP1*^−^*CD13^+^PDGFRA^+^ cells (**D**). (**E**) Images showing LRP1^+^CD13^+^PDGFRA^+^ and LRP1*^−^*CD13^+^PDGFRA^+^ cells on day 3 and day 7. 10× original magnification. (**F**) Quantitative measurement of the number of cells in fold-change relative to day 0 shows LRP1^+^CD13^+^PDGFRA^+^ cells have significantly higher cell numbers compared with LRP1*^−^*CD13^+^PDGFRA^+^ cells (*P* < 0.01) (*n* = 3). Data presented as mean ± SD. ***P < 0.001 by 2-tailed Student’s *t* test.

Given multiple progenitor clusters are present in the human periosteum (Figure 1G), we next determined the self-renewal and multilineage differentiation potentials of LRP1^+^CD13^+^PDGFRA^+^PDPN^+^ P-SSC cluster (cluster 1) compared with those of LRP1*^−^*CD13^+^PDPN*^−^* cells and LRP1*^−^*CD13^−^. After FACS isolation of LRP1^+^CD13^+^PDGFRA^+^PDPN^+^ P-SSCs and LRP1*^−^*CD13^+^PDPN*^−^*, or LRP1^−^CD13^−^ control cells from CD45^−^CD31^−^CD235a^−^ periosteal cells, an in vitro clonogenic assay revealed that LRP1^+^CD13^+^PDGFRA^+^PDPN^+^ P-SSCs had significantly greater viability and colony formation (Figure 4A). When each of those colonies was cultured at the same cell density (5 × 10^5^ cells/well), LRP1^+^CD13^+^PDGFRA^+^PDPN^+^ P-SSCs had ~2-fold more cell numbers at day 10, suggesting their higher proliferation capability (Figure 4B). In addition, an in vitro trilineage differentiation assay determined that the cells from LRP1^+^CD13^+^PDGFRA^+^PDPN^+^ P-SSC colonies were highly osteogenic (alizarin red staining) and chondrogenic (Alcian blue staining) but showed minimal adipogenic differentiation (Figure 4C). By contrast, LRP1*^−^*CD13^+^PDPN*^−^* cells had reduced osteogenic potential, whereas LRP1^−^CD13^−^ cells showed reduced differentiation potentials for all 3 lineages (osteogenic, chondrogenic, and adipogenic), indicating the roles of different subset of progenitor cells with distinct differentiation potentials in the human periosteum (Figure 4C). To assess the long-term repopulation ability of human P-SSCs, whole periosteal cells were cultured in serum-rich media and underwent multiple passages at weekly intervals. Consistently, FACS analysis of cells of each passage revealed LRP1^+^CD13^+^PDGFRA^+^ cells preserved their population, and the majority of these cells (~60%) expressed stem cell markers (CD73^+^CD164^+^), while only 10% of LRP1*^−^*CD13^+^PDGFRA^+^ cells expressed CD73 and CD164 (Figure 5, A–D) (1). Furthermore, LRP1^+^CD13^+^PDGFRA^+^ cells had a significantly greater rate of proliferation compared with LRP1*^−^*CD13^+^PDGFRA^+^ cells, indicating a critical role of LRP1 in stem and progenitor cell self-renewing and proliferative capacity in vitro (Figure 5, E and F).

### Human LRP1^+^CD13^+^PDGFRA^+^PDPN^+^ P-SSCs engraft into bone injury and contribute to bone healing in vivo.

**Figure 6:**
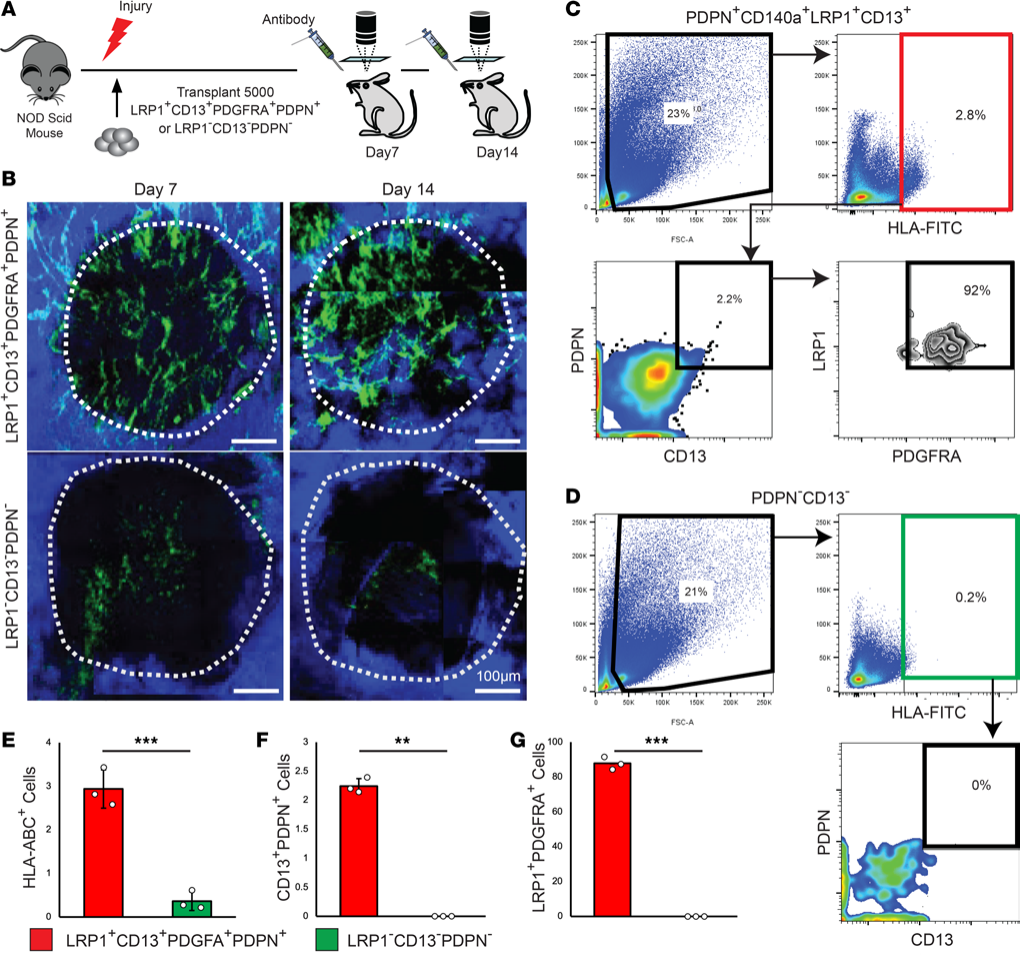
LRP1^+^CD13^+^PDGFRA^+^PDPN^+^ human P-SSCs engraft into calvarial bone injuries and contribute to bone healing in vivo. (**A** and **B**) Drill hole injuries were made on 12-week-old NOD *scid* mice (*n* = 3 per group), and 5,000 LRP1^+^CD13^+^PDGFRA^+^PDPN^+^ or 5,000 LRP1*^−^*CD13*^−^*PDPN*^−^* periosteal cells were FACS-sorted and transplanted. Seven and 14 days after transplantation, the transplanted cells were stained with HLA-ABC-FITC antibody and analyzed by in vivo intravital imaging. (**C** and **D**) On day 15 posttransplantation, transplanted LRP1^+^CD13^+^PDGFRA^+^PDPN^+^ (**C**) and LRP1*^−^*CD13*^−^*PDPN*^−^* (**D**) cells were FACS analyzed for their surface expression of HLA-ABC (**E**) and SSC markers, CD13^+^PDPN^+^ (**F**), and PDGFRA^+^LRP1^+^ (**G**). (**E** and **F**) Graphs show the percentage of the indicated cells from **C** and **D** (*n* = 3 mice per group). Data presented as mean ± SD. **P < 0.01, ***P < 0.001 by 2-tailed Student’s *t* test.

**Figure 7:**
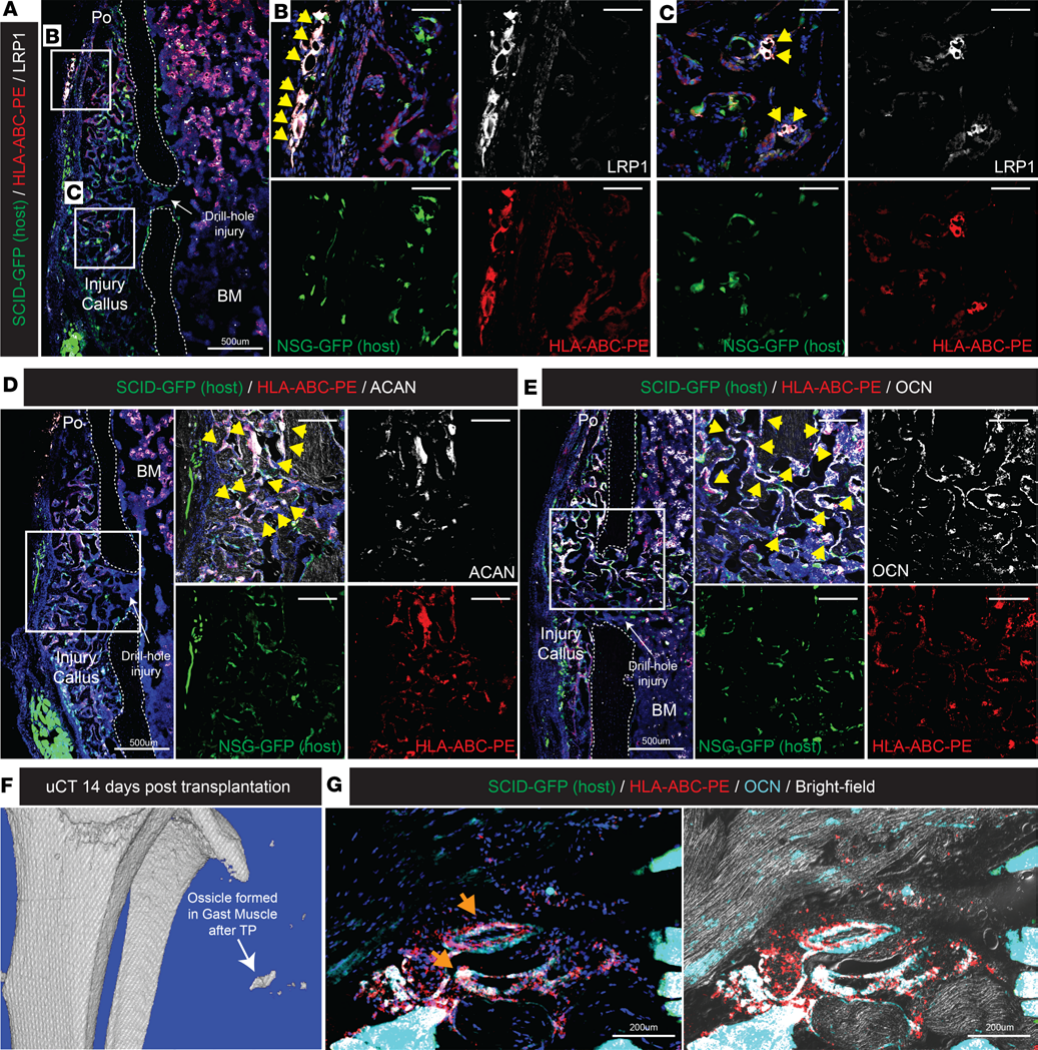
LRP1^+^CD13^+^ human P-SSCs engraft and possess osteochondrogenic differentiation potential in vivo. (**A**–**E**) Drill hole injury was made on 12-week-old NSG-EGFP (NOD *scid* IL2Rgamma^null^ CAG-EGFP) mouse tibia (*n* = 3 per group) and 10,000 LRP1^+^CD13^+^ periosteal cells were FACS-isolated and transplanted. Fourteen days after transplantation, animals were euthanized and tibia isolated for immunofluorescence staining for (**A**–**C**) HLA-ABC-PE, LRP1, (**D**) aggrecan (ACAN), and (**E**) osteocalcin (OCN) antibody. (HLA-ABC^+^LRP1^+^, HLA-ABC^+^Acan^+^, or HLA-ABC^+^Ocn^+^ cells are indicated with yellow arrow) (**F**) Hind limb 3D reconstructed μCT images of bone organoids derived from LRP1^+^CD13^+^ P-SSCs transplanted into the gastrocnemius muscle 14 days after transplantation. (**G**) Immunostaining of xenograft bone organoids derived from LRP1^+^CD13^+^ P-SSCs implanted into the gastrocnemius muscle of NSG-EGFP mouse hosts with indicated antibody staining (HLA-ABC^+^Ocn^+^ bone ossicles are indicated with orange arrow).

To better define the long-term repopulation and bone-forming ability of LRP1^+^CD13^+^PDGFRA^+^PDPN^+^ P-SSCs in vivo, we performed transplantation and consecutive intravital imaging of human P-SSCs at injury sites in the calvaria or long bone of immunocompromised mice (NOD *scid*). Although the transplantation of SSCs with a scaffold into the kidney capsule (5) or mammary fat pad (8) was commonly used to determine SSC repopulation and differentiation capabilities, we reasoned that a more physiologically and clinically relevant approach would be to transplant human P-SSCs onto a mouse periosteal injury site. A total of 5,000 FACS-sorted LRP1^+^CD13^+^PDGFRA^+^PDPN^+^ P-SSCs or LRP1*^−^*CD13*^−^*PDPN*^−^* control cells from the human periosteal CD45*^−^*CD31*^−^*CD235a*^−^* fraction were transplanted with Matrigel onto the drill hole calvarial injury site of NOD *scid* mice. To detect engrafted cells in living animals, we stained cells at the calvarial injury sites with human HLA-ABC-FITC antibody about 20 minutes prior to each imaging session. Using intravital imaging, we tracked successful engraftment and repopulation of LRP1^+^CD13^+^PDGFRA^+^PDPN^+^ P-SSCs 7 days after transplantation and their expansion 14 days after the transplantation (Figure 6, A and B). By contrast, only a few LRP1*^−^*CD13*^−^*PDPN*^−^* cells were engrafted at day 7, and even fewer cells were present 14 days after transplantation (Figure 6B). FACS analysis at 15 days after transplantation verified the number of donor cells present in the calvarial injury was over 10-fold higher with LRP1^+^CD13^+^PDGFRA^+^PDPN^+^ P-SSCs compared with LRP1*^−^*CD13*^−^*PDPN*^−^* cells (Figure 6, C–E, HLA-FITC). In addition, a subset (~2%) of P-SSCs maintained CD13 and PDPN expression; approximately 88% of these cells were PDGFRA and LRP1 positive, while none of the LRP1*^−^*CD13*^−^*PDPN*^−^* cells expressed these markers (Figure 6, F and G), implying a strong repopulation capability of LRP1^+^CD13^+^PDGFRA^+^PDPN^+^ P-SSCs in vivo. To further validate osteochondrogenic differentiation potential of P-SSCs in vivo, FACS-sorted LRP1^+^CD13^+^PDGFRA^+^PDPN^+^ P-SSCs were transplanted with Matrigel onto the drill hole defect sites of tibial injury or gastrocnemius muscle of NOD *scid* IL2Rgamma^null^ CAG-EGFP (NSG-EGFP) mice. To distinguish human donor cells from mouse recipient cells, immunofluorescence staining for HLA-ABC-PE, LRP1, ACAN, and OCN on tibial injury callus revealed coexpression of HLA-ABC and LRP1 (Figure 7, A–C). More importantly, these HLA-ABC^+^ human periosteal cells expressed ACAN and OCN, indicating their chondrogenic and osteogenic differentiation potentials, respectively (Figure 7, D and E). Consistent with our bone injury models, these human P-SSCs were able to form bone organoids following intramuscular transplantation in vivo (Figure 7, F and G). However, we did not observe such function in LRP1*^−^*CD13*^−^* cells (data not shown). These data suggest LRP1^+^CD13^+^PDGFRA^+^PDPN^+^ periosteal cells are, at least in part, long-term repopulating P-SSCs that can maintain their immunotypic markers and contribute to bone healing, even after transplantation onto the bone injury site in vivo.

### Mouse Prx1^+^ P-SSCs selectively express LRP1 and CD13.

**Figure 8:**
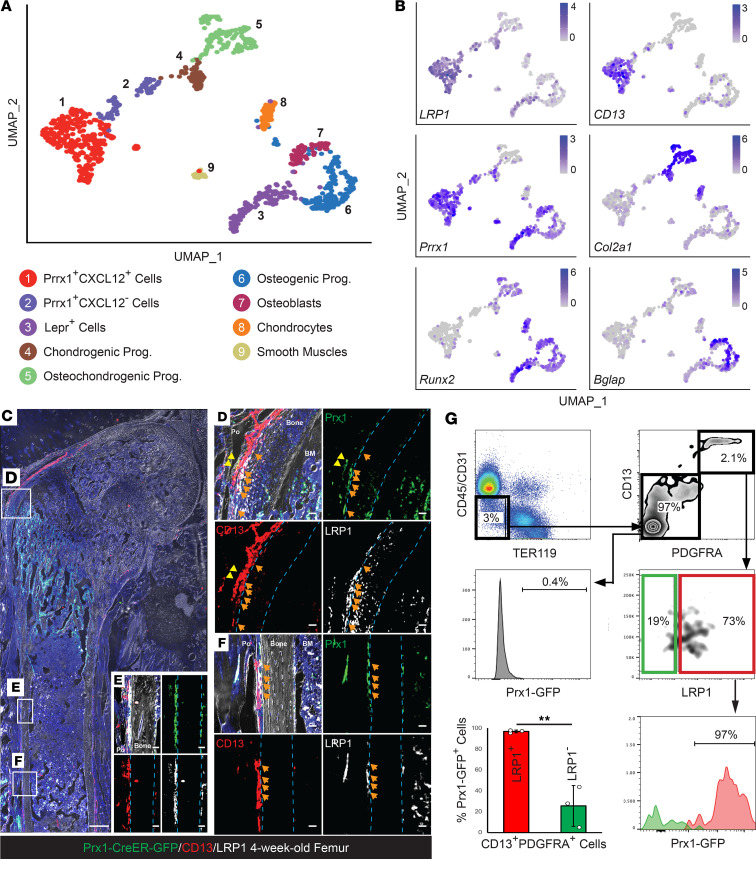
Mouse *Prx1*^+^ P-SSCs selectively express LRP1. (**A**) UMAP visualization of 9 different color-coded clusters, including *Prx1*^+^ P-SSCs (clusters 1 and 2) and other osteochondrogenic, smooth muscle cell clusters in long bone periosteal tissues in the mouse. (**B**) UMAP-based transcriptional plots of PRRX1, LRP1, CD13, osteogenic (Runx2 & Bglap), and chondrogenic (Col2a1) markers. (**C**–**F**) Representative immunofluorescence staining images of the indicated markers (Prx1-GFP, CD13 [Alexa-594], and LRP1 [Alexa-633]; GFP^+^CD13^+^LRP1^+^ cells: orange arrow; GFP^+^CD13*^+^*LRP1*^−^* cells: yellow arrowhead) of femoral sections (10x). (**G**) FACS analysis of surface expression of Prx1-GFP in LRP1^+^CD13^+^PDGFRA^+^ and LRP1*^−^*CD13^+^PDGFRA^+^ periosteal cells. Data presented as mean ± SD. **P < 0.01 by 2-tailed Student’s *t* test.

To determine whether LRP1 expression is conserved across the species, we collected FACS-isolated nonhematopoietic/nonendothelial cells (CD45*^−^*CD31*^−^*Ter119*^−^*) from mouse long bone periosteum and performed 10x scRNA-Seq. Subsequent UMAP clustering analysis after filtering out hematopoietic lineage cells identified 9 clusters within the mouse periosteal stromal cells, including *Prx1*^+^ P-SSCs (cluster 1 and 2), *Lepr*^+^ P-SSCs (cluster 3), osteogenic or chondrogenic cells (clusters 4–8), and smooth muscle cells (cluster 9) (Figure 8A). More importantly, we observed that mouse *Prx1*^+^ P-SSC clusters highly and selectively expressed LRP1 and CD13, while the expression of these markers was undetectable in both mature osteoblasts and chondrocytes (Figure 8B). Given GFP-expressing cells in *Prx1*^CreER-GFP^ transgenic mice are osteochondrogenic stem progenitor cells in the periosteum (12, 23), we further validated LRP1 and CD13 expression in *Prx1*^GFP+^ P-SSCs. Immunofluorescence staining of LRP1 and CD13 on the femoral section of 4-week-old *Prx1*^CreER-GFP^ mice revealed that the majority of *Prx1*^GFP+^ periosteal cells were colabeled with LRP1 and CD13 (Figure 8, C–F). Unexpectedly, we found many LRP1^+^ cells were present in BM perivascular regions. However, LRP1^+^ cells coexpressing CD13 were present only in the periosteal inner cambium layer, and the majority of these cells were *Prx1*^GFP^ positive (Figure 8, C–F). Moreover, FACS analysis of periosteal cells from 4-week-old *Prx1*^CreER-GFP^ mice revealed ~97% of LRP1^+^CD13^+^PDGFRA^+^ cells were *Prx1*^GFP+^, while only ~26% in LRP1*^−^*CD13^+^PDGFRA^+^ cells were *Prx1*^GFP+^ (Figure 8G). Taken together, these data indicate both human and mouse P-SSCs selectively express LRP1 and CD13, and LRP1^+^CD13^+^PDGFRA^+^ can be used as a combination of surface markers to isolate both human and mouse periosteal stem cell subsets.

### Loss of LRP1 in skeletal mesenchymal lineage leads to skeletal deformity.

**Figure 9:**
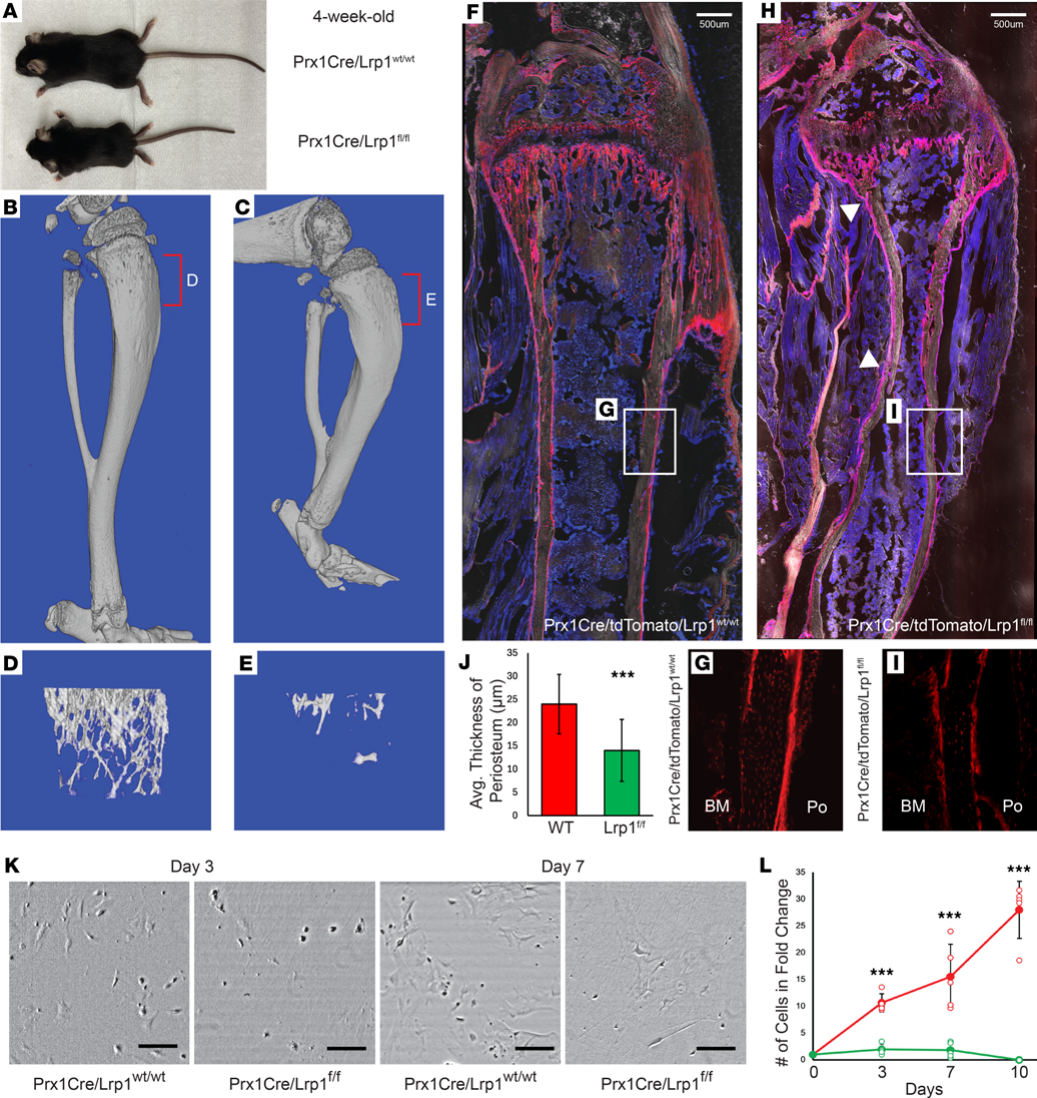
Conditional deletion of *Lrp1* in *Prx1*^+^ cells leads to skeletal dysplasia. (**A**–**C**) Representative images of 4-week-old *Prx1*^Cre^ *Lrp1^wt/wt^* and *Prx1*^Cre^ *Lrp1^fl/fl^* mice and tibial 3D reconstructed μCT images. (**D** and **E**) μCT images of trabecular bone regions. (**F**–**I**) Histological section of *Prx1*^Cre^ *Rosa26^tdTomato^ Lrp1^wt/wt^* and *Prx1*^Cre^ *Rosa26^tdTomato^ Lrp1^fl/fl^* mice and (**G** and **I**) zoomed-in regions of periosteum (microfracture: white arrowhead). (**J**) Average thickness of tdTomato^+^ periosteum from *Prx1*^Cre^ *Rosa26^tdTomato^ Lrp1^wt/wt^* and *Prx1*^Cre^ *Rosa26^tdTomato^ Lrp1^fl/fl^* mice. (**K** and **L**) Images showing *Prx1*^Cre^ *Lrp1^wt/wt^* and *Prx1*^Cre^ *Lrp1^fl/fl^* periosteal cells on day 3 and day 7. Quantitative measurement of number of cells in fold-change relative to day 0 shows *Prx1*^Cre^ *Lrp1^fl/fl^* periosteal cells have significantly lower cell numbers compared with *Prx1*^Cre^ *Lrp1^wt/wt^* mice (*P* < 0.01) (*n* = 5). Data presented as mean ± SD. ***P < 0.001 by 2-tailed Student’s *t* test.

Although recent studies reported bone deformities and developmental hip dysplasia in *Lrp1*-mutant or heterozygous knockout mice (27), the function of LRP1 in periosteal skeletal stem cells is essentially unknown. Given that our data showed a selective expression of LRP1 in both human and mouse P-SSCs, we tested how the conditional deletion of *Lrp1* in mouse skeletal stem and progenitor cells (*Prx1*^Cre^ *Lrp1^fl/fl^*) affects skeletal development. Notably, at 4 weeks of age, *Prx1*^Cre^ *Lrp1^fl/fl^* mice were substantially smaller than WT littermates and showed joint deformity along with impaired mobility (Figure 9A). Subsequent μCT analysis of 4-week-old *Prx1*^Cre^ *Lrp1^fl/fl^* mouse tibia revealed significantly shorter length, distorted shape, and reduced trabecular bone volume compared with WT littermates (Figure 9, B–E). Immunofluorescence imaging of *Prx1*^Cre^ *Rosa26*^tdTomato^ *Lrp1^fl/fl^* mouse tibia showed a bulged epiphysis, unusual curvature of the diaphysis, and regions of reduced marrow area. More importantly, we observed a thinner and uneven periosteum layer and microfracture (white arrowhead) in *Prx1*^Cre^ *Rosa26*^tdTomato^ *Lrp1^fl/fl^* mice, supporting a critical role of *LRP1* in periosteal maintenance (Figure 9, F–I). Next, to test whether *Lrp1* regulates the proliferation of periosteal progenitor cells in vitro, we isolated periosteal cells from *Prx1*^Cre^ *Lrp1^wt/wt^* and *Prx1*^Cre^ *Lrp1^fl/fl^* mice and cultured at the same density. We found significantly lower proliferative capability in *Prx1*^Cre^ *Lrp1^fl/fl^* periosteal cells, and these cells were not viable after 10 days of culture (Figure 9, K and L). These results suggest that *LRP1* plays an important role in bone and cartilage development (28) and periosteal cell regulation and maintenance.

### Local administration of LRP1 agonist promotes P-SSC proliferation and bone injury healing.

**Figure 10:**
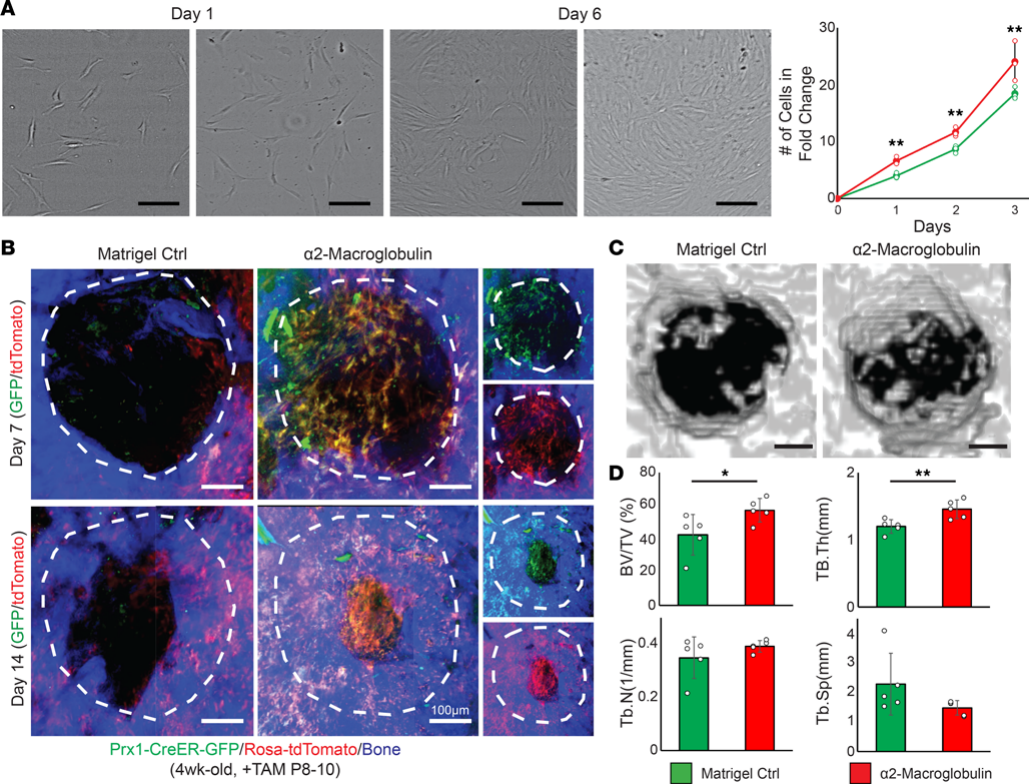
Treatment with LRP1 agonist promotes periosteal cell proliferation and bone injury healing. (**A**) Images showing human periosteal cells treated with control or LRP1 agonist α_2_-macroglobulin (α_2_M) (10 nM), on day 1 and day 6. Quantitative measurement of number of cells in fold-change relative to day 0 shows that the α_2_M-treated group has significantly higher cell numbers compared with the control (*P* < 0.01) (*n* = 3). 10x original magnification. (**B**) Calvarial injuries in *Prx1*^CreER-GFP^ *Rosa26*^tdTomato^ mice (4- to 5-week-old, tamoxifen at P8–P10) were treated with α_2_M mixed with Matrigel (2 μL at 10 ng/μL of Matrigel), or Matrigel alone (control), at days 0, 2, and 4. The presence of *Prx1*-GFP^+^ and Tomato^+^ cells and bone healing were assessed by sequential in vivo intravital imaging at days 7 and 14 after injury. Bone, blue (*n* = 3). (**C**) Representative μCT images of tibial drill hole injury after 7 days of treatment with Matrigel control (left) or α_2_M mixed with Matrigel (right) at days 0, 2, and 4. 10x original magnification. (**D**) Quantification of μCT scans showing bone volume/total volume (BV/TV), trabecular thickness (Tb.Th), trabecular number (Tb.N), and trabecular spacing (Tb.Sp) (*n* = 5). Data presented as mean ± SD. **P < 0.01 by 2-tailed Student’s *t* test.

Given that loss of *Lrp1* in mouse skeletal stem and progenitor lineage leads to early skeletal defects, we further tested whether LRP1 agonists can induce P-SSC proliferation in vitro and in vivo. When human P-SSCs were treated with an LRP1 agonist, α_2_-macroglobulin (α_2_M), we found a significant increase in their proliferation (Figure 10A). Next, to test whether α_2_M treatment induces P-SSCs in vivo, calvarial injuries were induced in 4- to 5-week-old *Prx1*^CreER-GFP^ *Rosa26*^tdTomato^ mice (P8–P10, tamoxifen^+^) and treated with α_2_M mixed with Matrigel (2 μL at 10 ng/μL), or Matrigel alone (2 μL, control), at the site of injury on days 0, 2, and 4. Intravital imaging of calvarial injury at day 7 and 14 showed a significant increase in the number of *Prx1*^GFP+^ Tomato^+^ cells in α_2_M-treated sites of injury (Figure 10B). In agreement with the calvarial injury response, we found that treatment with α_2_M mixed with Matrigel (2 μL at 10 ng/μL) at the tibial injury sites on days 0, 2, and 4 significantly increased bone volume and trabecular thickness compared with control injuries treated with Matrigel alone (Figure 10, C and D). These results suggest that early, local treatment of bone injuries with α_2_M is beneficial for increasing recruitment of endogenous P-SSCs, thus leading to bone healing.

## Discussion

The in vivo identity and molecular characteristics of human periosteal SSCs have long been controversial. Our single-cell analysis of the human periosteum revealed human periosteal cells are highly heterogeneous, and endogenous P-SSC subsets have distinct gene signatures with unique surface marker expression (LRP1^+^CD13^+^PDGFRA^+^PDPN^+^). More importantly, we identified a human P-SSC subpopulation (cluster 1) similarly express known SSC surface markers and stemness markers (*SOX4*, *GAS1*, and *DPP4*). The single-cell trajectory and RNA velocity analysis showed cluster 1 as the initial state of skeletal-lineage cells (Figure 1, D and E), demonstrating it is the apex of periosteal progenitor cell hierarchy and cluster 1 can differentiate into CPCs (cluster 3 and cluster 4), FAPs (cluster 6), and OPCs (cluster 8). However, whether all periosteal cells are derived from common P-SSC or from distinct P-SSC subsets still needs to be elucidated.

Previous studies showed P-SSCs in mice undergo osteochondrogenic differentiation without adipocyte differentiation in vivo (8, 13). However, it remains to be determined whether the limited adipogenesis of P-SSCs is due to cell-intrinsic mechanisms or due to environmental regulation. We revealed that human P-SSC clusters highly express early osteogenic and chondrogenic genes and regulators but do not express adipogenic genes (*ADIPOQ* [not expressed], *PPARG*, and *PRDM16*) (Supplemental Figure 2). Consistently, FACS-isolated human P-SSCs (LRP1^+^CD13^+^PDGFRA^+^PDPN^+^) have robust in vitro osteochondrogenic differentiation potentials with minimal adipogenic differentiation, supporting that cluster 1 can annotate endogenous human P-SSCs with high osteochondrogenic potentials.

Previous mouse studies identified multiple bone-forming stem/progenitor cell populations originating from different skeletal tissues (13). However, a detailed transcriptome analysis and population heterogeneity among these SSCs at the single-cell level are still unclear. More importantly, freshly isolated P-SSCs and BM-SSCs in humans have not been compared. Although the periosteal and BM-derived stromal cells originate from different individuals, with the adoption of batch effect correction (18), our studies showed for the first time to our knowledge evidence of distinct clusters from minimally manipulated human periosteal and BM stem/progenitor cells. Our studies also showed the P-SSC cluster (cluster 1) is molecularly distinct from BMSCs. Moreover, our results also revealed the human P-SSC cluster highly and selectively express LRP1. Although it has been reported that *Lrp1* deletion in *Runx2*^Cre^ and *Ctsk*^Cre^ mice decreases bone mass (29), the expression and function of LRP1 in human periosteal cells are largely unknown. By combining LRP1 and CD13 with previously identified SSC markers (PDGFRA and PDPN), we revealed that LRP1^+^CD13^+^PDGFRA^+^PDPN^+^ cells are exclusively present in human P-SSC clusters. More importantly, these cells exhibit high clonogenic capacity and can undergo osteochondrogenic differentiation in vitro, with known stem cell surface marker expression. In addition, these cells are long-term repopulating cells even after multiple rounds of serial passages, suggesting their self-renewing capacity. Similarly, the *Prx1*^+^ P-SSC cluster derived from mouse long bone also have a higher expression of LRP1. In particular, coexpression of LRP1 and CD13 in *Prx1*^GFP+^ cells occurs in long bone periosteum, and approximately 97% of LRP1^+^CD13^+^PDGFRA^+^ periosteal cells are *Prx1*^GFP+^, as determined from FACS analysis, further supporting the combination of LRP1 and CD13 as a selective marker for the subset of human and mouse P-SSC populations.

Functional analysis of human SSCs is technically challenging. Human cell transplantation in mouse kidney capsules is a common method; however, human P-SSC transplantation onto a periosteal injury site is more physiologically and clinically relevant compared with the kidney capsule transplantation of SSCs. Our approach with the successful engraftment and robust repopulation of LRP1^+^CD13^+^PDGFRA^+^PDPN^+^ cells, when transplanted into calvaria or tibia injury sites of immunocompromised mice, supports the in vivo SSC proliferative capability. Additionally, we revealed that LRP1^+^CD13^+^PDGFRA^+^PDPN^+^ P-SSCs were able to contribute to bone injury healing and differentiated into osteochondrogenic cells in vivo. The presence of LRP1^+^CD13^+^PDGFRA^+^PDPN^+^ cells in calvaria and tibia injury sites after 14 days of transplantation and forming bone organoids in xenograft system further supports that these cells are P-SSCs capable of self-renewal, proliferation, and differentiation in vivo. More importantly, conditional deletion of *Lrp1* in mouse skeletal stem and progenitor cells led to a severe bone phenotype with compromised periosteal cell proliferative capability in vitro, suggesting a critical role of LRP1 in periosteal cell function and skeletal maintenance. By contrast, α_2_M, an LRP1 agonist, treatment enhanced human P-SSC proliferation in vitro. Last, local administration of α_2_M promotes P-SSC–mediated bone injury healing, suggesting that LRP1 is a novel positive regulator of P-SSCs. Future studies will be needed to define the environmental regulation of LRP1 in P-SSCs and other periosteal progenitor populations.

One limitation of the current study is that obtaining intact human periosteum from exactly the same physiological location is challenging, and thus our samples may contain P-SSC population that are undergoing bone injury repair process or subset of SSCs from perichondrium. In addition, α_2_M inactivates multiple proteinases and binds to variety of growth factors, so the impact of α_2_M-induced bone healing may not be solely due to activation of LRP1^+^ P-SSCs. However, to our knowledge, this is the first study to show the molecular characteristics of endogenous human periosteal cells by scRNA-Seq and to identify P-SSC clusters with osteochondrogenic potentials. Furthermore, we identified periosteal stem cell markers, LRP1 and CD13, which in combination can selectively isolate human and mouse P-SSCs, and a function of LRP1 in P-SSC proliferation and differentiation in vivo aiding in bone healing. These results will facilitate the characterization of normal and diseased P-SSCs and the development of novel cell therapies to promote endogenous human P-SSCs for bone regeneration and repair.

## Methods

### Sex as a biological variable.

Our study contained samples from both men and women. In all reported data, sex was not considered as a biological variable, and findings for both sexes were similar.

### Preparation of single-cell suspensions from human periosteal sample.

Human periosteal tissues were obtained from 8 patients (mean age, 51 ± 12 years) when undergoing fracture surgery. Collected tissues were then transferred to MEMα medium containing 10% fetal bovine serum (FBS) on ice, and the overlying fascia and muscle were carefully removed. The tissues were fragmented with a scalpel into small pieces and washed with phosphate-buffered saline (PBS), then resuspended in pre-warmed PBS containing 2% FBS and 5 mg/mL collagenase type I (MilliporeSigma, C0130). The samples were incubated at 37°C for 1 hour with gentle shaking. Samples were centrifuged at 350*g* for 5 minutes, then resuspended in PBS containing 2% FBS, 5 mg/mL collagenase type I, and 2 mg/mL dispase. The samples were additionally incubated at 37°C for 1 hour with gentle shaking. Digestion was terminated by adding MEMα medium with 10% FBS. The digested samples were filtered through a 70 μm, and then a 40 μm, strainer to remove any remaining debris. After centrifugation at 350*g* for 5 minutes, the collected cells were resuspended in FACS buffer (1× PBS with 1% FBS).

### Animals.

Four- to 5-week-old and 8-week-old C57BL/6 (The Jackson Laboratory [Jax] strain 000664), *Prx1*^CreER-GFP^ (Jax strain 029211), Rosa26-*loxP*-stop-*loxP*-tdTomato (Jax strain 007909), NOD.Cg-Prkdc^scid^/J (Jax strain 001303), and NOD.Cg-Prkdc^scid^ Il2rg^tm1Wjl^ Tg(CAG-EGFP)10sb/SzJ (Jax strain 021937) mice were purchased. For *Prx1*^CreER^ induction, mice were injected intraperitoneally with 50 mg/kg of tamoxifen (Santa Cruz Biotechnology, CAS 10540-29-1) P8–P10.

### Isolation of mouse periosteal cells.

To isolate periosteal cells, the dissected femurs and tibias from mice were placed in PBS, and the overlying skin, fascia, and muscle were carefully removed. The bones with periosteum were incubated in ice-cold PBS with 1% FBS for 30 minutes, and the loosely associated periosteum was peeled off using a scalpel, forceps, and dissecting scissors. The soft floating periosteal tissues collected with a 40 μm strainer were then incubated with 5–10 mL of 0.2% collagenase and 10% FBS in PBS at 37°C for 1 hour, and the dissociated periosteal cells were washed with PBS, filtered with a 40 μm strainer, and resuspended at approximately 1 × 10^7^ cells/mL.

### Tibial injury and μCT analysis.

All tibial injuries were performed using aseptic technique. A small (<1 cm) incision was made on the anterior side of the hind limb below the knee to expose the proximal tibia. A 26s-gauge needle was used to create an injury approximately 0.5 mm in diameter. The skin was closed using sutures, and a small amount of triple antibiotic ointment was applied to the surrounding skin and sutures using a sterile cotton swab. For the treatment of tibial injuries with α_2_M (Innovative Research, IMSA2M100UG), 8-week-old female WT C57BL/6 mice were treated topically with α_2_M (2 μL at 10 ng/μL Matrigel) or under control conditions (2 μL Matrigel) on days 0, 2, and 4 after injury. Injury healing was assessed 7 days after injury.

Tibial cortical bone healing was assessed using μCT (μCT 40, SCANCO Medical AG). Scans were performed using an x-ray setting of 55 kVp voltage and 145 μA current with a 200 ms exposure at high resolution. Calibration images were collected prior to data acquisition. Scans were performed with an effective voxel size of 10 μm^3^. For image analysis, a global upper threshold of 255 and lower threshold of 120 (μCT gray scale value) were used for all samples to separate the bone from the soft tissue. CTan version 1.14.1 (SkyScan, Bruker-microCT) was used to determine bone volume over total volume.

### Flow cytometry.

The following antibodies were used for human cells: CD45-Pacific blue (clone: HI30, BioLegend, 304021), CD31-Pacific blue (clone: WM59, BioLegend 303113), CD235a-Pacific blue (clone: HI264, BioLegend, 349107), CD13-Brilliant Violet 605 (clone: WM15, BioLegend, 301727), CD13-PE (clone: WM15, BioLegend, 301703), Podoplanin-APC-CY7 (clone: NC-08, BioLegend, 337029), PDGFRA-PE-CY7 (clone: 16A1, BioLegend, 323507), LRP1-PE (clone: A2MR-α2, BD Biosciences, 550497), CD73-Brilliant Violet 605 (clone: AD2, BioLegend, 344023), CD164-FITC (clone: 67D2, BioLegend, 324805), and HLA-ABC-FITC (clone: W6/32, Invitrogen, 11-9983-42). The following antibodies were used for mouse periosteal cells: CD45-Pacific blue (clone: 30-F11, BioLegend, 103125), CD31-eFluor 450 (clone: 390, Invitrogen, 48-0311-82), Ter119-APC-CY7 (clone: TER-119, BioLegend, 116223), PDGFRA-PE-CY7 (clone: APA5, BioLegend, 135911), CD13-PE (clone: R3-242, BD Biosciences, 558745), and LRP1 (clone: SA0290, Invitrogen, MA5-31959). Cells were stained in sorting buffer for 30 minutes at 4°C, washed once, and resuspended in sorting buffer. Flow cytometry experiments and sorting were performed using the LSRII and FACSAria cytometer (BD Biosciences). Data were analyzed with FlowJo software (TreeStar) and represented as histograms, contour plots, or dot plots for fluorescence intensity.

### 10x Genomics scRNA-Seq.

Following human periosteal cell isolation, lineage-positive cells were depleted using the MACS lineage cell depletion kit (Miltenyi Biotec, 130-092-211) to obtain lineage-negative cells. Lineage-negative periosteal cells were then FACS-sorted based on CD45, CD31, CD235a, and DAPI staining. Mouse periosteal cells were FACS-sorted based on CD45, CD31, TER-119, and DAPI staining. Trypan blue stain was used to assess cell viability and number; cells were resuspended at 1,000 cells/μL and loaded on a 10x Genomics Chromium single-cell controller to generate single-cell gel beads-in-emulsion. scRNA-Seq libraries were constructed using the Chromium Single Cell 3′ v3.1 (10x Genomics, PN-11000121) Reagent Kit, according to the manufacturer’s protocol. Amplified cDNA and final libraries were evaluated on an Agilent BioAnalyzer using a High Sensitivity DNA Kit (Agilent Technologies). Libraries were then sequenced with a 28–10–10–90 paired-end read, generating 300 million reads per sample on the Illumina NovaSeq 6000 analyzer.

### Pre-processing of scRNA-Seq data.

scRNA-Seq data were demultiplexed, barcode-processed, and aligned to the human genome, version GRCh38, using Cell Ranger (version 6.1.2) with default parameters. Further downstream analysis steps were performed using the Seurat R package (version 4.1.1). For quality control, we excluded cells with fewer than 500 detected genes and UMI counts per cell and more than 10% of mitochondrial read content. To integrate and apply correct batch effects among samples, we performed canonical correlation analysis (CCA) implemented in Seurat and used the 3,000 most variable genes for each dataset to identify anchors (30). Using anchors and corresponding scores, each dataset was integrated and used for dimensionality reduction and clustering subsequently.

### Dimensionality reduction and clustering.

We performed principal component analysis (PCA) and reduced the data to the top 15 PCA components and used UMAP on whole datasets. We used graph-based clustering of the integrated datasets with the Louvain algorithm after computing the shared nearest neighbor graph (31).

### DEG analysis.

We used the Wilcoxon rank-sum test to find DEGs in each cluster. DEGs were filtered by a minimum log_2_ fold-change for an average expression of 0.25, with a *P* value adjusted by Bonferroni’s correction using all genes in the dataset.

### RNA velocity.

The matrices for spliced and unspliced transcripts were constructed using the velocyto (version 0.17.17) python package with a human genome reference. The output loom files were used to compute RNA velocity and dynamics of splicing kinetics with the scVelo python package (version 0.2.4) dynamic modeling (19). Pseudotime trajectory analysis was generated based on RNA velocity to predict ancestors of individual cells, and the partition-based graph abstractions graph was generated (22).

### Comparative analysis of periosteal and BM aspirate concentrates.

For the comparative analysis between data sets for periosteal and BM aspirate concentrates, 2 sets of raw data for BM aspirate concentrates from National Center for Biotechnology (NCBI) Gene Expression Omnibus (GEO) GSE162692 were collected (14). The same filtering criteria from periosteal data were applied (number of genes detected and UMI counts per cells > 500; mitochondrial read counts < 10%). Datasets were integrated with CCA methods as described in *Pre-processing of scRNA-Seq data*.

### Gene set enrichment analysis.

We performed gene set enrichment analysis with the clusterProfiler package (4.10.0) to identify GO terms for biological processes (32). The enrichment score (ES) corresponds to a weighted Kolmogorov-Smirnov–like statistic (33). The *P* value of the ES is calculated using a permutation test. When the entire gene sets are evaluated, the estimated significance level is adjusted to account for multiple-hypothesis testing, and *q* values are calculated for FDR control.

### Immunofluorescence.

Frozen sections from human periosteal tissues that were fixed overnight in 4% paraformaldehyde were stained with primary rabbit monoclonal anti-CD13 antibody (Abcam, ab108310), rat monoclonal anti-podoplanin antibody (Invitrogen, 14-9381-82), and mouse anti-CD91 [LRP1] antibody (BD Biosciences, 550497) according to the manufacturers’ instructions. Similarly, frozen sections from mouse tibia, fixed in 4% paraformaldehyde and decalcified, were stained with rabbit monoclonal anti-LRP1 antibody (Invitrogen, MA5-31959), rabbit polyclonal anti-osteocalcin antibody (Abcam, ab198228), rabbit monoclonal anti-aggrecan antibody (Abcam, ab186414), and HLA-ABC-PE antibody (Invitrogen, 12-9983-42) according to the manufacturers’ instructions. Anti-rabbit Alexa Fluor 488 (Invitrogen, A-21206), anti-rabbit Alexa Fluor 594 (Invitrogen, A-11012 or A-21207), and anti-rat or anti-rabbit Alexa Fluor 647 (Invitrogen, A-21094 or A-32733) were used as secondary antibodies. VECTASHIELD (Vector Laboratories, H-1500) containing DAPI nuclear counterstain was used to mount the sections. Images were acquired with a Nikon A1-Rs confocal laser scanning microscope in the Integrated Microscopy Core at Baylor College of Medicine (BCM). Images were processed with the Nikon NIS-Elements software.

### Cell transplantation and live animal imaging after transplantation in vivo.

Human periosteal cell isolation and antibody staining for FACS were performed as stated above. Two populations of periosteal cells were collected: 1) CD45*^−^*CD31*^−^*CD235a*^−^* CD13^+^PDPN^+^PDGFRA^+^LRP1^+^ and 2) CD45*^−^*CD31*^−^*CD235a*^−^*CD13*^−^*PDPN*^−^*. Drill hole injuries were made to the calvaria of NOD *scid* (NOD.Cg-Prkdc^scid^/J, Jax strain 001303) mice with a 22G needle, near the intersection of sagittal and coronal sutures, and then approximately 5,000 cells in Matrigel (10 μL) were transplanted into the injury sites. Intravital microscopy was used 7 and 14 days after transplantation to confirm the engraftment of human periosteal cells. For live in vivo imaging of transplanted cells at injury sites, mice were anesthetized and prepared for visualization under a customized 2-photon and confocal hybrid microscope (Leica TCS SP8MP with DM6000CFS) specifically designed for live animal imaging, as described in our previous report (34). Prior to imaging, injury sites were stained with primary mouse monoclonal anti-human HLA-ABC FITC antibody (Invitrogen, 11-9983-42, 1:20) for 20 minutes in the dark, then washed with PBS. The mice were then mounted on a 3D axis motorized stage (Anaheim Automation), and the calvarial bones and injuries were visualized by second harmonic generation (440 nm SHG by femto-second titanium/sapphire laser pulses: 880 nm) from bone collagen fibers to identify the injury sites. FITC-expressing cells (488 nm excitation, 505–550 nm detection) were simultaneously imaged using confocal spectral fluorescence. Each image was recorded by *Z*-stacks with 50–100 μm depth from the bone surface at a 4 to 5 μm intervals. A phase coherence imaging–based image capture board (Snapper, Active Silicon) was used to acquire up to 3 channels simultaneously using the Leica Application Suite software (Version 3.3). Following in vivo imaging, the scalp was closed and sutured using a VICRYL plus suture (Ethicon), and postoperative care of mice was provided as previously described (34). The 3D images were reconstructed using the ImageJ software (NIH, Version 2.3.0/1.53q).

### Statistics.

Analyses were performed using GraphPad Prism 9.0 software. For comparison of 3 groups, differences were analyzed by 1-way ANOVA with a significant main effect of *P* < 0.05, followed by a Dunnett’s multiple-comparison test to detect differences between groups at a significance of *P* < 0.05. Differences between 2 experimental groups were determined with an unpaired 2-tailed *t* test at a significance of *P* < 0.05. Outcomes are reported as means ± SD. Number of samples per group is indicated in the figure legends.

### Study approval.

All human periosteal tissues were collected as part of a research study approved by the Institutional Review Board of BCM (H-40670) following written informed consent. All mice were maintained in pathogen-free conditions, and all procedures were approved by BCM’s Institutional Animal Care and Use Committee.

### Data availability.

Mouse and human scRNA-Seq data are available in the NCBI’s GEO database with the accession numbers GSE276574 and GSE278165, respectively. Values for all data points in graphs are reported in the Supporting Data Values file.

## Author contributions

YJ, LD, and DP conceived the study. YJ, LD, LO, and JRD developed methodology. YJ, LD, LO, KL, and DP investigated. YJ and LD performed data visualization. DP supervised. YJ, LD, and DP wrote the original draft. YJ, LD, and DP reviewed and edited the manuscript.

## Supplementary Material

Supplemental data

Supporting data values

## Version 1 10/15/2024

In-Press Preview

## Version 2 11/22/2024

Electronic publication
