# Supplementary material for: Identification of LRP1+CD13+ human periosteal stem cells that require LRP1 for bone repair
Source: JCI Insight. 2024 Nov 22;9(22):e173831. doi: 10.1172/jci.insight.173831 (PMC11601900; doi:10.1172/jci.insight.173831)
Supplement: Supplemental data [file jciinsight-9-173831-s011.pdf]

A

Sample Number	Age	Sex	Anatomical Location
Sample1 (scRNAseq)	34	F	Medial Malleolus
Sample2 (scRNAseq)	39	M	Medial Malleolus
Sample3 (scRNAseq)	52	F	Olecranon
Sample4 (scRNAseq)	40	M	Distal Radius
BM (scRNAseq)	66	M	Femoral Neck
Histology Section	58	F	Medial Malleolus
Transplantation (Cal)	58	F	Medial Malleolus
Transplantation (LB)	61	F	Lateral Malleolus
In vitro Culture	58/61	F	Lateral Malleolus/Medial Malleolus

B

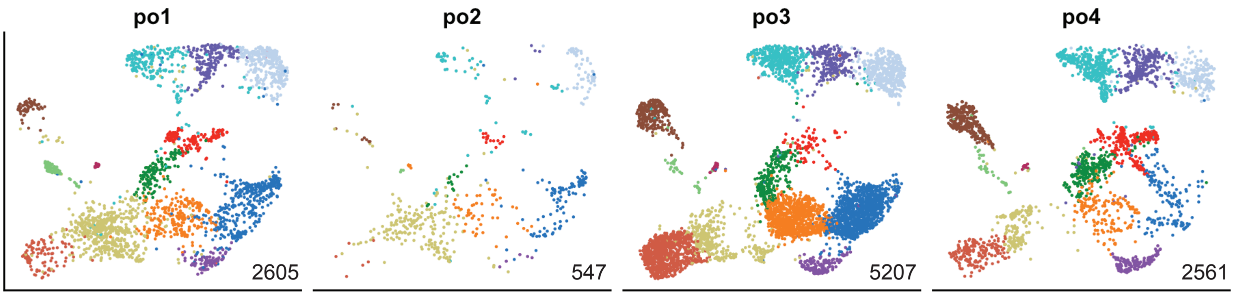

Figure S1.(A) Human periosteal cell donor age, sex, and anatomical location. (B) UMAP plots showing from four different samples.

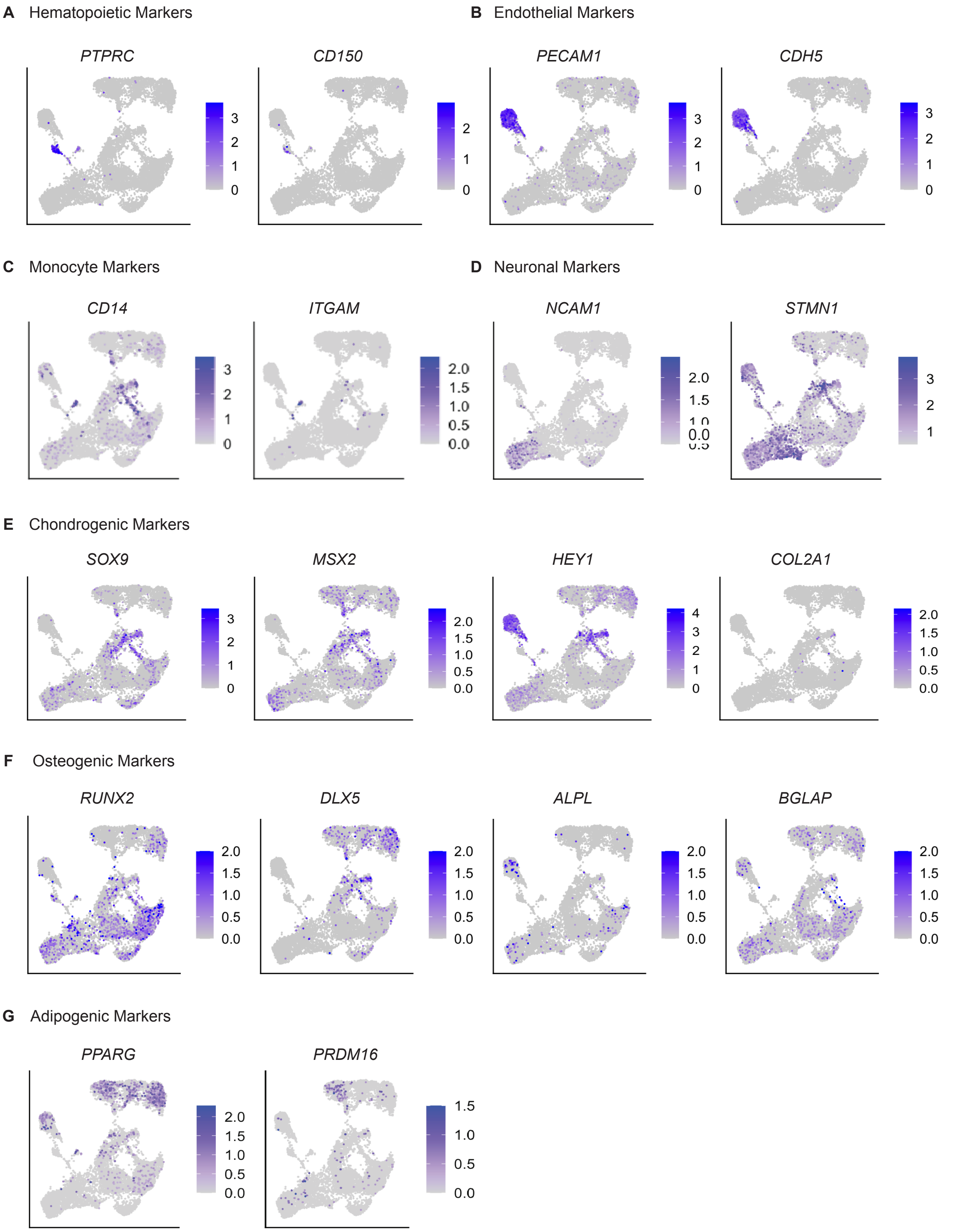

Figure S2. UMAP of representative human (A) Hematopoeitic, (B) Endothelial, (C) Monocyte, (D) Neuronal, (E) Chondrogenic, (F) Osteogenic, and (G) Adipogenic gene markers.

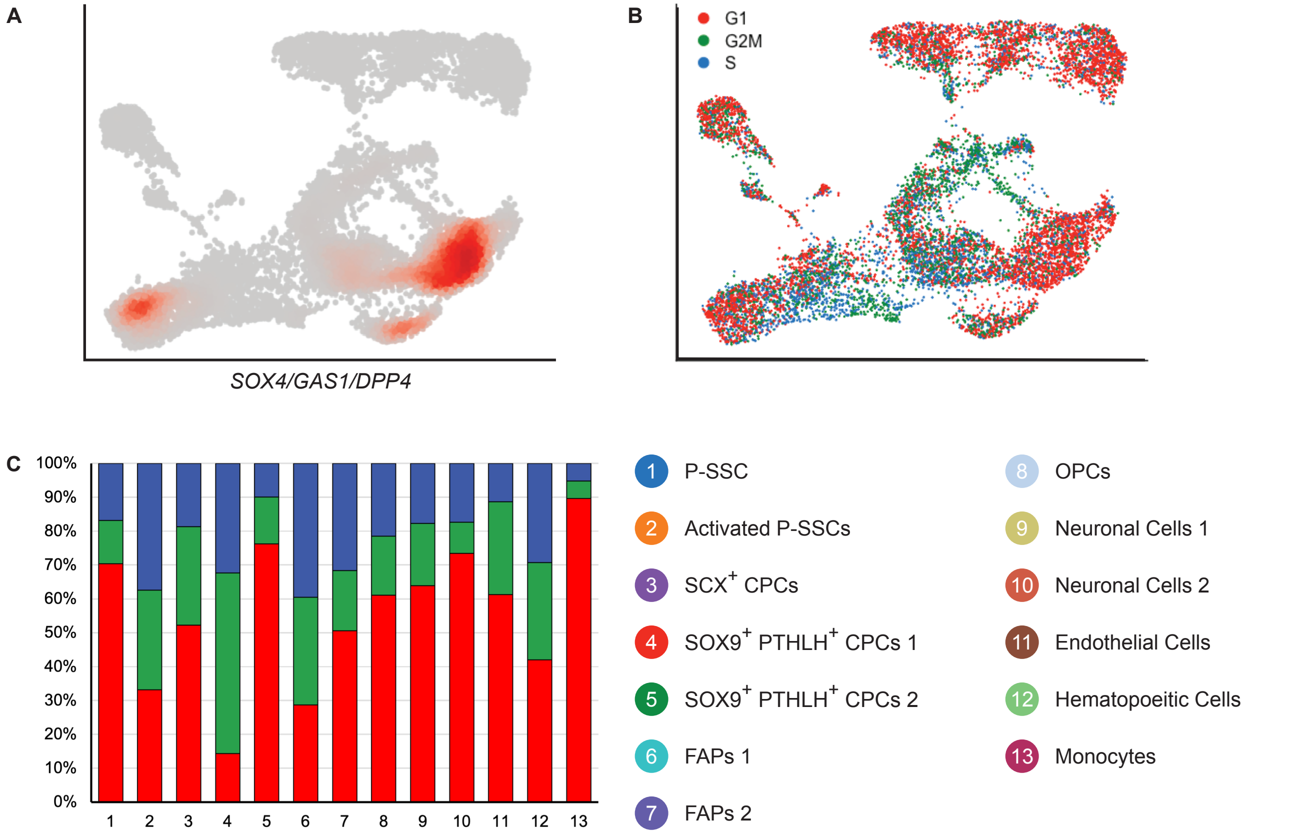

Figure S3. (A) UMAP joint densit plots of stemness markers. (B-C) Cell cycle phase distribution in periosteal clusters.

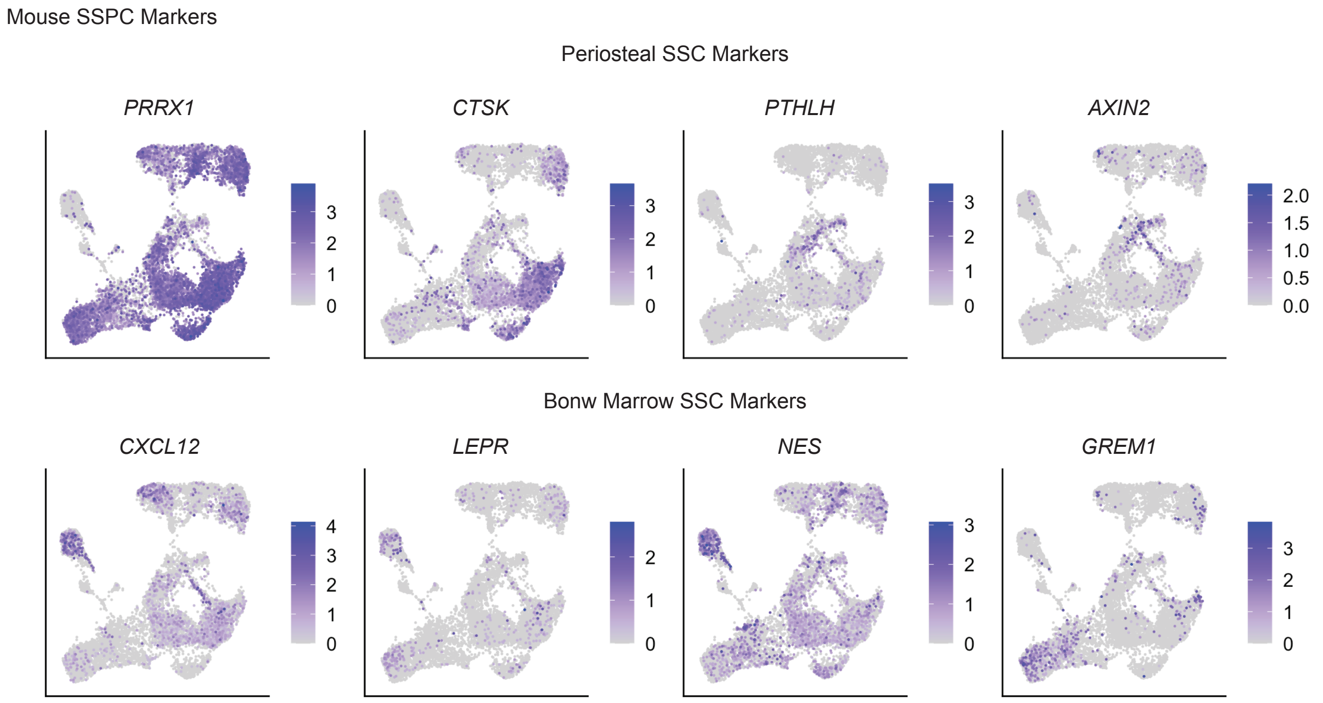

Figure S4. UMAP of representative mouse skeletal stem and progenitor cell gene expression markers.
